# Two-Stage Multiarmed Bandit for Reconfigurable Intelligent Surface Aided Millimeter Wave Communications

**DOI:** 10.3390/s22062179

**Published:** 2022-03-10

**Authors:** Ehab Mahmoud Mohamed, Sherief Hashima, Kohei Hatano, Saud Alhajaj Aldossari

**Affiliations:** 1 Electrical Engineering Department, College of Engineering at Wadi Addwasir, Prince Sattam Bin Abdulaziz University, Wadi Addwasir 11991, Saudi Arabia; s.alhajaj@psau.edu.sa; 2 Electrical Engineering Department, Faculty of Engineering, Aswan University, Aswan 81542, Egypt; 3 Computational Learning Theory Team, RIKEN-Advanced Intelligent Project, Fukuoka 819-0395, Japan; sherief.hashima@riken.jp (S.H.); hatano@inf.kyushu-u.ac.jp (K.H.); 4 Engineering and Scientific Equipment’s Department, Egyptian Atomic Energy Authority, Cairo 13759, Egypt; 5 Faculty of Arts and Science, Kyushu University, Fukuoka 819-0395, Japan

**Keywords:** millimeter wave, reconfigurable intelligent surface, multiarmed bandit, Thompson sampling, upper confidence bound

## Abstract

A reconfigurable intelligent surface (RIS) is a promising technology that can extend short-range millimeter wave (mmWave) communications coverage. However, phase shifts (PSs) of both mmWave transmitter (TX) and RIS antenna elements need to be optimally adjusted to effectively cover a mmWave user. This paper proposes codebook-based phase shifters for mmWave TX and RIS to overcome the difficulty of estimating their mmWave channel state information (CSI). Moreover, to adjust the PSs of both, an online learning approach in the form of a multiarmed bandit (MAB) game is suggested, where a nested two-stage stochastic MAB strategy is proposed. In the proposed strategy, the PS vector of the mmWave TX is adjusted in the first MAB stage. Based on it, the PS vector of the RIS is calibrated in the second stage and vice versa over the time horizon. Hence, we leverage and implement two standard MAB algorithms, namely Thompson sampling (TS) and upper confidence bound (UCB). Simulation results confirm the superior performance of the proposed nested two-stage MAB strategy; in particular, the nested two-stage TS nearly matches the optimal performance.

## 1. Introduction

A reconfigurable intelligent surface (RIS) is a promising technology to extend the coverage of the communication systems by means of passive antenna arrays [1]. This can be done by configuring the phase shifts (PSs) of the antenna elements to reflect the incoming electromagnetic wave towards an intended destination. Compared with the conventional amplify and forward (AF) and decode and forward (DF) relays, RIS has the advantages of low cost and ease of installation as no RF chains are needed [2]. Millimeter wave (mmWave) communication, i.e., 30~300 GHz band, is another promising technology for fifth-generation (5G) wireless communications and beyond due to its vacant frequencies enabling multi-Gbps transmissions [3,4,5,6]. However, due to its high operating frequencies, mmWave is characterized by a short-range transmission with increased susceptibility to path blockage [7]. This necessitates the use of directional antennas in the form of antenna beamforming training (BT) [8,9,10,11]. 

A symbiotic relationship exists between both technologies. On one side, RIS is considered an efficient solution for mmWave challenges, where RIS can extend the mmWave coverage and route around blockages. On the other side, mmWave can directly tune its beam direction towards the RIS location, and the RIS reflects this beam towards the intended mmWave receiver (RX) via adjusting its PSs. However, jointly optimizing the PSs of both mmWave transmitter (TX) and RIS antenna elements is challenging due to the complex estimation of the massive mmWave channel state information (CSI) at both RIS and mmWave users. Moreover, as RIS is entirely passive, it does not support any channel estimation operations. 

In this paper, for practical realization of the mmWave–RIS communication system and to avoid the estimation of the massive mmWave CSI, antenna codebooks are suggested for both mmWave TX and RIS. Without loss of generality, we follow the mmWave codebook design given in [12,13], where the codebooks are generated with 90-degree phase resolution without amplitude adjustment. We choose this codebook design due to its simplicity and standardization by mmWave standards, i.e., Wireless Gigabit (WiGig) standard [12,13]. Furthermore, to maximize the spectral efficiency at the intended mmWave user, PS vectors of both mmWave TX and RIS should be jointly optimized. However, this will consume a considerable beamforming training (BT) overhead, especially when using many PS vectors of sizeable antenna arrays. To efficiently address this problem, an online learning approach is proposed using a single-player multiarmed bandit (MAB) game. 

MAB is an online learning strategy where an agent tries to maximize its profit via playing over the bandit’s available arms. The agent attempts to compromise between consistently exploiting the arm, giving the maximum profit so far, or exploring new ones, known as *exploitation–exploration* trade-off. Based on the reward’s distribution, MAB games can be categorized as *stochastic* or *adversarial* MAB, where in the former the rewards come from a pre-known distribution while in the latter the rewards come arbitrarily. A complete survey of the MAB approach, including its categories and algorithms, can be found in [14,15]. In the formulated MAB game, the mmWave TX will be the player, the available space of the candidate PS vectors will be the arms of the bandit game, and the achievable spectral efficiency at the mmWave user will be the reward. Thus, one set of joint PS vectors can be tested at a time, which highly relaxes the BT overhead. Furthermore, a nested two-stage MAB methodology is proposed to reduce the complexity and increase the convergence rate of the proposed MAB game. Thus, the main contributions of this paper can be summarized as follows:

- The RIS-assisted mmWave communication system is considered, where an optimization problem is formulated to jointly adjust the PS vectors of both mmWave TX and RIS.
- Discrete PSs in the form of codebook design are suggested to relax the complicated CSI estimation problem at both mmWave TX and RIS. In this design, the PSs are assumed to be generated with 90-degree phase resolution with constant amplitude like the codebook design used by WiGig standards [12,13].
- A stochastic single-player MAB game is constructed to jointly optimize the PS vectors of mmWave TX and RIS. This facilitates the adjustment of both PS vectors successively in a time-by-time fashion, which highly reduces the required BT overhead. Typically, the only available information for a MAB player is its reward observation, without any details about the environment. Thus, considering mmWave PSs optimization as a MAB game eliminates the need for CSI estimation as the observed achievable spectral efficiency, i.e., the reward of the game, is the only needed information. This information can be easily obtained via the feedback channel between the mmWave RX and TX. Moreover, the suggested codebook design facilitates the implementation of the MAB game, where the PS vectors are considered as its arms. To reduce the complexity of the arm optimization as we have two sets of arms, one belonging to the mmWave TX and the other to the RIS, a nested two-stage MAB game is proposed in this paper. In this approach, the PS vector of mmWave TX is adjusted in the first MAB stage, and based on it, the PS vector of the RIS is modified in the next stage and vice versa over the time horizon. Thus, at each trial, the player needs to only explore one set of the PS vectors, either that belonging to the mmWave TX or that belonging to the RIS, which reduces the computational complexity of the constructed MAB game. Two common MAB algorithms, namely Thompson sampling (TS) [16] and upper confidence bound (UCB) [17], are used to implement the proposed nested two-stage MAB and compare their performances under the mmWave–RIS environment.
- Numerical analyses are conducted to prove the effectiveness of the proposed mmWave–RIS communication system over benchmarks against the optimal performance under different simulation scenarios.

The rest of this paper is constructed as follows: Section 2 summarizes the related works, and Section 3 discusses the system model and the problem formulation. Section 4 proposes the antenna codebook design and the nested two-stage MAB approach. Section 5 delivers the numerical analysis, followed by the concluding remarks in Section 6.

## 2. Literature Review

One way to overcome the continuously increasing capacity in the wireless communication systems is to control the channel itself to develop an intelligent radio environment besides other existing solutions (diversity, high-frequency waves, etc.). RIS is a programmable arrangement that controls the propagation of electromagnetic waves by varying its surface’s electric and magnetic characteristics. Furthermore, RIS can sense the radio environment by installing intelligent surfaces within the wireless system environment, which entirely or partially controls the features of the radio channels. Hence, RIS-assisted systems can improve the reliability and energy efficiency of wireless systems [1]. Lately, RIS has drawn much consideration as an up-and-coming technology that can suit future wireless systems demands [18], i.e., 6G and beyond. Hence, RIS has promoted wireless applications such as RIS-aided wireless power transfer [19], RIS-aided mobile edge computing [20], RIS-aided physical layer security [21,22], RIS-aided UAV communications [23,24], and mobility and handover management for RIS-aided wireless communications in high-speed trains [25].

There are limited related research works investigating the impact of RIS deployment in mmWave networks. A general tractable model for the coverage performance of the RIS-assisted mmWave networks focused on RIS and base station (BS) densities using stochastic geometry was proposed in [26]. A privacy-preserving design paradigm combining federated learning (FL) with RIS in the mmWave communication system was proposed in [27]. A deep learning algorithm was proposed in [28] to set up a relation between CSI and RIS configurations for better optimal communication rate performance. An efficient cascaded channel estimation model for an RIS-assisted mmWave MIMO system, with the wideband effect on the transmission model, was considered in [29]. A hybrid precoding (HP) design for the RIS-aided multiuser (MU) mmWave communication systems was investigated in [30]. Artificial intelligence (AI)-empowered mmWave communications, especially using RIS, were studied in [31]. To the best of our knowledge, all the current research works on RIS-assisted mmWave assume perfect CSI information. Based on it, the PS vectors of BS and RIS are adjusted to maximize the achievable spectral efficiency at the RX. Without this CSI information, these PS vectors cannot be optimized due to the assumption of continuous PS. However, perfect CSI is a strong assumption violating the RIS hypothesis of being utterly passive without any channel estimation functionality.

## 3. System Model

**Figure 1:**
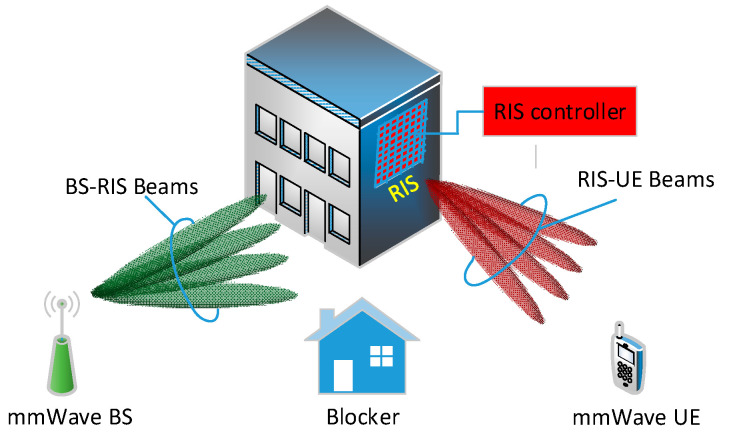
RIS-assisted mmWave communication system.

Figure 1 shows the system model of the RIS-assisted mmWave communication, where RIS is used to connect the mmWave BS with a single-antenna mmWave user equipment (UE) by routing around the blocker. RIS is equipped with a uniform planner array (UPA) of *M* antenna elements, while mmWave BS is equipped with a uniform linear array (ULA) of *N* antenna elements. An RIS controller is used to control the PSs of RIS antenna elements based on the selected PS vector. In addition, mmWave BS and RIS are connected through a dedicated communication link for controlling and information exchange. As a result, the received signal at the UE can be expressed as follows:

(1) $$x=h_{RU}^{H}\Phi_{i}H_{BR}f_{j}s+n,\;$$

In (1), s is the transmitted symbol, and x is the received one where $E\left[ss^{H}\right]=P$, and P is the TX power. ${\left(.\right)}^{H}$ means Hermitian transpose and $n\sim CN\left(0,\sigma_{0}^{2}\right)$ is the complex additive white Gaussian noise (AWGN) with zero mean and variance of $\sigma_{0}^{2}$. $f_{j}\in {\mathbb{C}}^{N\times 1}$ is the analog precoder vector of size N×1 applied at the mmWave BS, and $\Phi_{i}\in {\mathbb{C}}^{M\times M}$ is a diagonal matrix of size M×M containing the PSs of the RIS antenna elements in its diagonal. {i,j: 1≤i≤|ℛ|, 1≤j≤|ℱ|} are the indices of the used Φ and f, where ℛ and ℱ are their finite sets. $H_{BR}\in {\mathbb{C}}^{M\times N}$ is the channel matrix of size M×N between BS and RIS, while $h_{RU}\in {\mathbb{C}}^{M\times 1}$ is the channel vector of size M×1 between the RIS and UE. Following the geometric channel models with limited scatterers given in [30], $H_{BR}$ and $h_{RU}$ can be expressed as follows:

(2) $$H_{BR}=\sqrt{\frac{MN}{L_{BR}}}\overset{L_{BR}}{\underset{l=1}{\sum }}\xi_{l}\Lambda_{R}\left(\chi_{l}^{\left(AoA\right)},\delta_{l}^{\left(AoA\right)}\;\right)\Lambda_{B}\left(\chi_{l}^{\left(AoD\right)}\;\right),\;$$

(3) $$h_{RU}=\sqrt{\frac{M}{L_{RU}}}\overset{L_{RU}}{\underset{l=1}{\sum }}\nu_{l}\Lambda_{R}\left(\theta_{l}^{\left(AoD\right)},\phi_{l}^{\left(AoD\right)}\;\right),\;$$

where $L_{BR}$ and $L_{RU}$ are the number of channel paths between BS and RIS and between RIS and UE, respectively. $\xi_{l}\sim CN\left(0,\sigma_{\xi_{l}}^{2}\right)$ and $\nu_{l}\sim CN\left(0,\sigma_{\nu_{l}}^{2}\right)$ are the complex path gains of the l-th path in $L_{BR}$ and $L_{RU}$, respectively. $\Lambda_{R}\left(\chi_{l}^{\left(AoA\right)},\delta_{l}^{\left(AoA\right)}\;\right)$, and $\Lambda_{B}\left(\chi_{l}^{\left(AoD\right)}\;\right)$ are the response vectors of the l-th path array at the RIS and BS, where $\chi_{l}^{\left(AoA\right)}\left(\delta_{l}^{\left(AoA\right)}\right)$ and $\chi_{l}^{\left(AoD\right)}\;$ are the azimuth (elevation) angle of arrival (AoA) and angle of departure (AoD), respectively. In addition, $\Lambda_{R}\left(\theta_{l}^{\left(AoD\right)},\phi_{l}^{\left(AoD\right)}\;\right)$ is the response vector of the *l*-th path at the RIS, where $\theta_{l}^{\left(AoD\right)}$ and $\phi_{l}^{\left(AoD\right)}$ are the corresponding azimuth and elevation AoD. Generally, for any θ and ϕ, $\Lambda_{R}\left(\theta ,\phi \;\right)$ can be expressed as follows [30]:

(4) $$\Lambda_{R}\left(\theta ,\phi \;\right)=\frac{1}{\sqrt{M}}{\left[1,\ldots ,\;e^{j\frac{2\pi }{\lambda }d\left(p\sin\left(\theta \right)+q\cos\left(\phi \right)\right)},\ldots \right]}^{T},\;$$

where d is the antenna spacing and λ is the carrier wavelength and $0\leq \left\{p,q\right\}\leq \left(\sqrt{M}-1\right)$. By analogy, $\Lambda_{B}\left(\chi_{l}^{\left(AoD\right)}\;\right)$ is defined as follows [30]:

(5) $$\Lambda_{B}\left(\theta \;\right)=\frac{1}{\sqrt{N}}{\left[1,\ldots ,\;e^{j\frac{2\pi }{\lambda }dn\sin\left(\chi_{l}^{\left(AoD\right)}\right)},\ldots \right]}^{T},\;$$

where 0≤n≤(N−1).

The mmWave–RIS optimization problem aims to jointly optimize Φ and f for maximizing the achievable spectral efficiency ψ in bps/Hz at the UE. Mathematically speaking this can be expressed as follows:

(6) $$\left\{i^{*},\;j^{*}\right\}=\underset{i,j}{\max}\left(\psi_{\Phi_{i}f_{j}}\right),\;$$

where

$$\psi_{\Phi_{i}f_{j}}=\log_{2}\left(1+\frac{P\left(h_{RU}^{H}\Phi_{i}H_{BR}f_{j}\right){\left(h_{RU}^{H}\Phi_{i}H_{BR}f_{j}\right)}^{H}}{\sigma_{0}^{2}}\right)$$

Herein, $\psi_{\Phi_{i}f_{j}}$ is the spectral efficiency at the UE resulting from using $\Phi_{i}$ and $f_{j}$, and the indices of the optimal values of Φ and f are represented by $\left\{i^{*},\;j^{*}\right\}$. Most of the existing literature assumes perfect CSI information; i.e., $H_{BR}$ and $h_{RU}$ are well known at both BS and RIS. Based on that, both Φ and f can be jointly adjusted using different iterative techniques [30,31,32]. However, this is a strong assumption as it is too difficult to estimate $H_{BR}$ and $h_{RU}$ due to the use of massive antenna elements at both BS and RIS. Furthermore, RIS should be utterly passive without any channel estimation functionality.

## 4. Proposed Antenna Codebook and MAB Approach 

In this section, antenna codebook and MAB approach are suggested for the mmWave–RIS system to overcome mmWave CSI estimation and jointly adjust the PS vectors of BS and RIS.

### 4.1. Antenna Codebook Design 

To eliminate the need for CSI estimation, discrete PSs are considered for both mmWave BS and RIS, where they constitute the antenna codebook of both. In this context, we will utilize the antenna codebook of WiGig standards for PS design at both BS and RIS [12,13]. This codebook-based beam switching involves fixed beam patterns and can be realized using a predefined pool of antenna weight vectors maintained at TX and RX. Columns of a codebook matrix specify the beamforming weight vector that corresponds to a unique beam pattern. The TX–RX beam pattern pair that optimizes a certain cost function is searched during beamforming according to an agreed criterion. Codebooks support a variety of antenna array geometries and offer flexibility in terms of the number, size, and spacing between antenna elements. For phased array antennas, the columns of the codebook matrix specify the discrete PSs applied to individual antenna elements. The patterns may be generated without amplitude adjustment to obtain processing power savings. As a guiding principle, the columns of the codebook are made orthogonal to each other so that multiple beams can be generated simultaneously without significant interference. These beams can also be synthesized to create a wider beam. Thus, in this codebook design, the PS vectors for K≤A, where A is the total number of antenna elements and K is the total number of PS vectors (i.e., beam directions), are given by column vectors of the following matrix:

(7) $$V\left(a,k\right)=j^{\mathrm{floor}\left\{\frac{a\times \mathrm{mod}\left(k+K/2,K\right)}{K/4}\right\}},\;$$

a=0,…, A−1, k=0,…, K−1 

In the case that K=M/2, the PS vector at k=0 becomes

(8) $$V\left(a,0\right)={\left(-j\right)}^{\mathrm{mod}\left(a,K\right)},\;a=0,\ldots ,\;A-1\;$$

Thus, the columns in V are the available space for constructing f and the diagonal of Φ.

### 4.2. Proposed Nested Two-Stage MAB Approach

Jointly optimizing the values of Φ and f using the prementioned codebook design will consume a considerable BT overhead due to the search over |ℛ||ℱ| different candidate beams. Instead, an online learning approach is proposed to successively obtain the optimal solution over the time horizon. This results in considerably reducing the BT overhead as only one pair $\Phi_{i}$ and $f_{j}$ will be tested at a time. In this context, an online single-player MAB game is constructed to address this problem efficiently. In this formulation, the BS is considered as the player of the bandit game; the available joint values of $\Phi_{i}$ and $f_{j}$ are the arms of the bandit; and the achievable spectral efficiency at the UE, i.e., $\psi_{\Phi_{i}f_{j}}$, is the reward. This MAB-based optimization problem can be mathematically formulated as follows:

(9) $$\underset{I\left(1\right),\ldots ,I\left(T_{H}\right)}{\max}\frac{1}{T_{H}}\underset{t}{\sum }\underset{i,j\;}{\sum }I_{i_{t}j_{t}}\left(\psi_{\Phi_{i_{t}}f_{j_{t}}}\right)\;,$$

s.t.

(1) $T_{H}\in \left(0,\;Z^{+}\right),$
(2) $\underset{i,j}{\sum }I_{i_{t}j_{t}}=1,$

where $T_{H}$ indicates the time horizon and $Z^{+}$ is the set of all positive integers. $\psi_{\Phi_{i_{t}}f_{j_{t}}}$ is the spectral efficiency resulting from using $\Phi_{i}$ and $f_{j}$ combination at time t, i.e., $\Phi_{i_{t}}$ and $f_{j_{t}}$. $I_{i_{t}j_{t}}$ is a selection indicator, which is equal to 1 if the combination $\Phi_{i}$ and $f_{j}$ is selected at time t and 0 otherwise. The constraint $\underset{i,j}{\sum }I_{i_{t}j_{t}}=1$ means that only one Φ and f combination is allowed to be selected at time t. To reduce the computational complexity of the constructed MAB game, a nested two-stage MAB strategy is proposed. In the proposed algorithm, the value of Φ is adjusted in the first MAB stage for a particular value of f. Then, based on the adjusted value of Φ, the value of f is adjusted in the second MAB stage, and so on over the time horizon. In this context, two common MAB algorithms are proposed to implement the suggested nested two-stage MAB approach, namely TS and UCB algorithms.

#### 4.2.1. Proposed Nested Two-Stage TS Algorithm

TS is based on a pure Bayesian strategy [16], where prior/posterior distributions are considered for the arms’ rewards. The parameters of the assumed probabilistic model are initialized for each arm at the beginning of the algorithm. Then, random samples are taken from the constructed distributions, and the arm related to the highest random sample is selected and played. After obtaining the rewards corresponding to the played arm, its parameters are updated for the posterior distribution of the next round of the MAB game. In the proposed TS algorithm, normal distributions are considered for the spectral efficiency corresponding to each value of Φ and f at time t, i.e., $\psi_{\Phi_{i_{t}}f_{j_{t}}}$, where 1≤i≤|ℛ|, 1≤j≤|ℱ|. This means that $\psi_{\Phi_{i_{t}}f_{j_{t}}}\sim \;N\left({\bar{\psi }}_{\Phi_{i_{t}}f_{j_{t}}},\sigma_{\Phi_{i_{t}}f_{j_{t}}}^{2}\right)$, where ${\bar{\psi }}_{\Phi_{i_{t}}f_{j_{t}}}$ and $\sigma_{\Phi_{i_{t}}f_{j_{t}}}^{2}$ are the mean and variance of the assumed normal distribution, and ${\bar{\psi }}_{\Phi_{i_{t}}f_{j_{t}}}$ is the average value of $\psi_{\Phi_{i_{t}}f_{j_{t}}}$. This assumption comes from the AWGN term given in (1). Algorithm 1 gives the proposed nested two-stage TS algorithm, where the inputs to the algorithm are the spaces of codebooks ℛ. and ℱ and the outputs are the adjusted values of $\Phi_{i^{*}}$ and $f_{j^{*}}$. At the beginning of the algorithm, i.e., t=1 the average spectral efficiencies ${\bar{\psi }}_{\Phi_{i_{t}}f_{j_{t}}}$, the variances $\sigma_{\Phi_{i_{t}}f_{j_{t}}}^{2}$, and the number of selections $Z_{\Phi_{i_{t}}f_{j_{t}}}$ corresponding to all values of $\Phi_{i}$ and $f_{j}$. are set to 0, 1, and 0, respectively. In addition, a PS vector f, i.e., $f_{j_{t}^{*}}$, is initialized by picking it uniformly from its corresponding PS codebook. For $2\leq t\leq T_{H}$, where $T_{H}$ is the total time horizon, nested two-stage TS algorithms are performed as follows:

**Algorithm 1.** Nested Two-Stage TS Algorithm **Output:**$\Phi_{i^{*}}$ and $f_{j^{*}}$
**Input:**ℛ, ℱ** Initialization**: At t=1,
     1. ${\bar{\psi }}_{\Phi_{i_{t}}f_{j_{t}}}=0$, $\sigma_{\Phi_{i_{t}}f_{j_{t}}}^{2}=1$, $Z_{\Phi_{i_{t}}f_{j_{t}}}=0$, 1≤i≤|ℛ|, 1≤j≤|ℱ|
     2. Select a value of $f_{j_{t}^{*}}\;$ at random from its finite set ℱ
 **For** $t=2,\;\ldots .,\;T_{H}$   
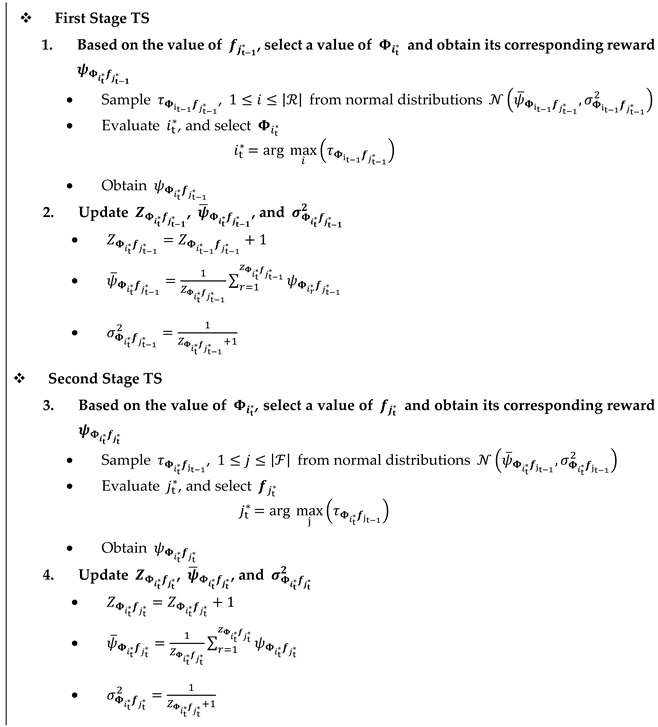
 **END For**


In the first stage and based on the value of $f_{j_{t-1}^{*}}$, a value of $\Phi_{i_{t}^{*}}$ is selected and its corresponding reward $\psi_{\Phi_{i_{t}^{*}}f_{j_{t-1}^{*}}}$ is obtained. This is done by sampling the prior distributions of $\psi_{\Phi_{i_{t-1}}f_{j_{t-1}^{*}}}$, i.e., $\tau_{\Phi_{i_{t-1}}f_{j_{t-1}^{*}}}\sim \;N\left({\bar{\psi }}_{\Phi_{i_{t-1}}f_{j_{t-1}^{*}}},\sigma_{\Phi_{i_{t-1}}f_{j_{t-1}^{*}}}^{2}\right),\;1\leq i\leq \left|ℛ\right|$. Then, the index 

$i_{t}^{*}$ corresponding to the maximum $\tau_{\Phi_{i_{t-1}}f_{j_{t-1}^{*}}}$ is selected as follows:

(10) $$i_{t}^{*}=\underset{\;}{\arg\;\underset{i}{\max}\left(\tau_{\Phi_{i_{t-1}}f_{j_{t-1}^{*}}}\right),\;}\;$$

Next, the value of Φ matrix corresponding to this index, i.e., $\Phi_{i_{t}^{*}}$, is obtained. Afterward, its corresponding reward $\psi_{\Phi_{i_{t}^{*}}f_{j_{t}^{*}}}$ is achieved, and its model parameters $Z_{\Phi_{i_{t}^{*}}f_{j_{t-1}^{*}}}$, ${\bar{\psi }}_{\Phi_{i_{t}^{*}}f_{j_{t-1}^{*}}}$, and $\sigma_{\Phi_{i_{t}^{*}}f_{j_{t-1}^{*}}}^{2}$ are updated for its posterior distribution as given in Algorithm 1, where the methodology presented in [33] is used for updating ${\bar{\psi }}_{\Phi_{i_{t}^{*}}f_{j_{t-1}^{*}}}$ and $\sigma_{\Phi_{i_{t}^{*}}f_{j_{t-1}^{*}}}^{2}$.

In the second stage TS and based on $\Phi_{i_{t}^{*}}$ coming from the first stage, a value of $f_{j_{t}^{*}}$ is adjusted, and its corresponding reward $\psi_{\Phi_{i_{t}^{*}}f_{j_{t}^{*}}}$ is obtained. In this procedure, random samples $\tau_{\Phi_{i_{t}^{*}}f_{j_{t-1}}}\sim \;N\left({\bar{\psi }}_{\Phi_{i_{t}^{*}}f_{j_{t-1}}},\sigma_{\Phi_{i_{t}^{*}}f_{j_{t-1}}}^{2}\right)$, 1≤j≤|ℱ|, are taken, and the index $j_{t}^{*}$ corresponding to the maximum sample value is chosen as follows:

(11) $$j_{t}^{*}=\underset{\;}{\arg\;\underset{j}{\max}\left(\tau_{\Phi_{i_{t}^{*}}f_{j_{t-1}}}\right)},\;$$

Again, the value of f matrix corresponding to this index, i.e., $f_{j_{t}^{*}}$, is obtained. Then, its corresponding reward $\psi_{\Phi_{i_{t}^{*}}f_{j_{t}^{*}}}$ is achieved, and its model parameters are updated for the posterior distribution of the next round of the MAB game as given in Algorithm 1.

#### 4.2.2. Proposed Nested Two-Stage UCB Algorithm 

UCB is based on increasing the confidence of the chosen arm by decreasing its uncertainty. This is done by exploiting the arm with the maximum achievable average reward so far while exploring the less selected ones. Algorithm 2 summarizes the proposed nested two-stage UCB algorithm. Like the TS algorithm, the inputs to the algorithm are the spaces of codebooks ℛ and ℱ, and the outputs are the adjusted values of $\Phi_{i^{*}}$ and $f_{j^{*}}$. For initialization, at t=1, the average spectral efficiencies ${\bar{\psi }}_{\Phi_{i_{t}}f_{j_{t}}}$ corresponding to all values of $\Phi_{i}$. and $f_{j}$ are set to uniform random values in the range of [0, 1], where 1≤i≤|ℛ|, 1≤j≤|ℱ|, and their corresponding numbers of selections, i.e., $Z_{\Phi_{i_{t}}f_{j_{t}}}$, are set to 1. Moreover, a PS vector f, i.e., $f_{j_{t}^{*}}$, is picked uniformly from its corresponding PS codebook, i.e., ℱ. For $2\leq t\leq T_{H}$, nested two-stage UCB algorithms are conducted as follows: 

In the first UCB stage, based on the value of $f_{j_{t-1}^{*}}$ and index of the Φ matrix, $i_{t}^{*}$ is selected based on the UCB policy as follows [17]:

(12) $$i_{t}^{*}=\underset{\;}{\arg\;\underset{i}{\max}\left({\bar{\psi }}_{\Phi_{i_{t-1}}f_{j_{t-1}^{*}}}+\sqrt{\frac{2\ln\left(t\right)}{Z_{\Phi_{i_{t-1}}f_{j_{t-1}^{*}}}}}\right)\;}$$

where ${\bar{\psi }}_{\Phi_{i_{t-1}}f_{j_{t-1}^{*}}}$ represents the exploitation term, while the term $\sqrt{\frac{2\ln\left(t\right)}{Z_{\Phi_{i_{t-1}}f_{j_{t-1}^{*}}}}}$ represents the exploration term of the UCB policy. After selecting $\Phi_{i_{t}^{*}}$, its corresponding reward $\psi_{\Phi_{i_{t}^{*}}f_{j_{t-1}^{*}}}$ is obtained and its related parameters $Z_{\Phi_{i_{t}^{*}}f_{j_{t-1}^{*}}}$ and ${\bar{\psi }}_{\Phi_{i_{t}^{*}}f_{j_{t-1}^{*}}}$ are updated as given in Algorithm 2. Based on the selected value of $\Phi_{i_{t}^{*}}$, the value of $f_{j_{t}^{*}}$ is adjusted in a nested manner via the second stage UCB as given in Algorithm 2. Then, its corresponding reward $\psi_{\Phi_{i_{t}^{*}}f_{j_{t}^{*}}}$ is obtained and its related parameters $Z_{\Phi_{i_{t}^{*}}f_{j_{t}^{*}}}$ and ${\bar{\psi }}_{\Phi_{i_{t}^{*}}f_{j_{t}^{*}}}$ are updated as given in Algorithm 2.

**Algorithm 2.** Nested Two-Stage UCB Algorithm **Output:**$\Phi_{i^{*}}$ and $f_{j^{*}}$
**Input:**ℛ, ℱ** Initialization**: At t=1
    1. Set
${\bar{\psi }}_{\Phi_{i_{t}}f_{j_{t}}}$ based on uniform random in the range [0, 1], and $Z_{\Phi_{i_{t}}f_{j_{t}}}\;$= 1, 1≤i≤|ℛ|, 1≤j≤|ℱ|
    2. Select a value of $f_{j_{t}^{*}}\;$ at random
** For** $t=2,\;\ldots .,\;T_{H}$   
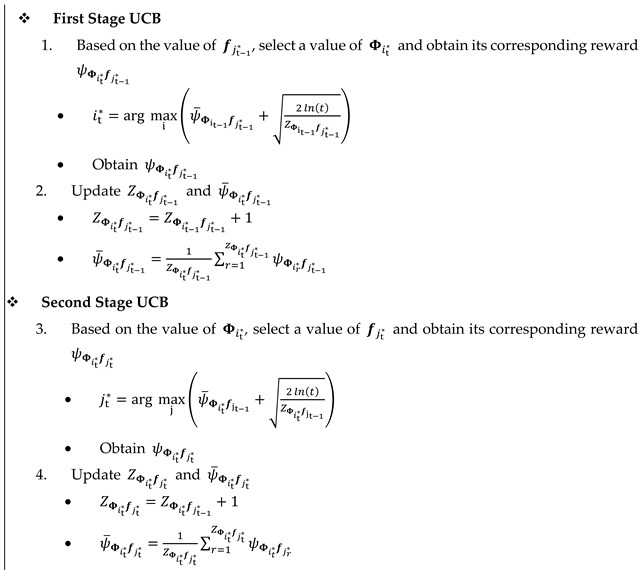
 **END For**


## 5. Numerical Analysis

In this section, Monto Carlo (MC) numerical simulations are conducted to prove the effectiveness of the proposed nested two-stage MAB algorithms compared to the random PS selection, where values of Φ and f are picked uniformly, against the optimal performance. The optimal performance is obtained by testing all available candidate pairs of Φ and f and selecting the best one maximizing ψ. Table 1 summarizes the utilized simulation parameters unless otherwise stated. In addition, it is assumed that the line-of-sight (LoS) path is 10 dB higher than the other paths [34].

**Figure 2:**
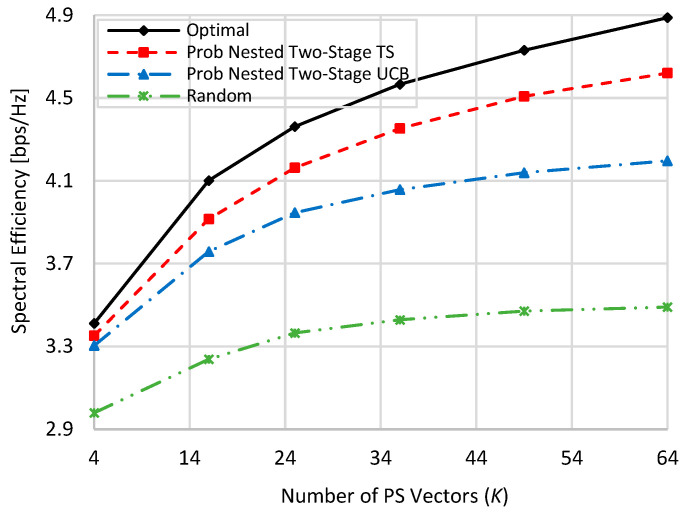
Spectral efficiency against the used number of beams when *M = N = K* at zero blockage.

Figure 2 shows the spectral efficiency performances of the compared schemes i.e., nested two-stage TS, UCB, and random at no blockage against the used number of PS vectors (*K*), where *K* = *M* = *N*. Generally speaking, as the number of *K* increases, the spectral efficiencies of all schemes increase due to the increase in the received power affected by the increment in the beamforming gain. Although the proposed nested two-stage MAB algorithms do not need CSI estimation and only use the observed spectral efficiency, they have good performances against the optimal performance compared to random PS selection. Moreover, the proposed nested two-stage TS algorithm outperforms all other compared schemes due to its Bayesian policy, which constructs prior/posterior distributions to the achievable spectral efficiency. As the assumed normal distribution highly matches the actual distribution of the attainable spectral efficiency, the proposed nested two-stage TS outperforms UCB-based one. Random PS selection shows the worst performance because it selects the PS vectors arbitrarily without any optimization objective. At *K* = 4, about 98.5%, 97%, and 85.3% of the optimal performance are obtained by the proposed nested two-stage TS, UCB, and random selection, respectively. These values become 94.3%, 86%, and 71.7% when *K* = 64, where the number of alternative beam pairs is highly increased. As TS is a Bayesian-based approach, its performance is still near the optimal one, while the performance of the other two schemes is highly degraded compared to the case of *K* = 4.

**Figure 3:**
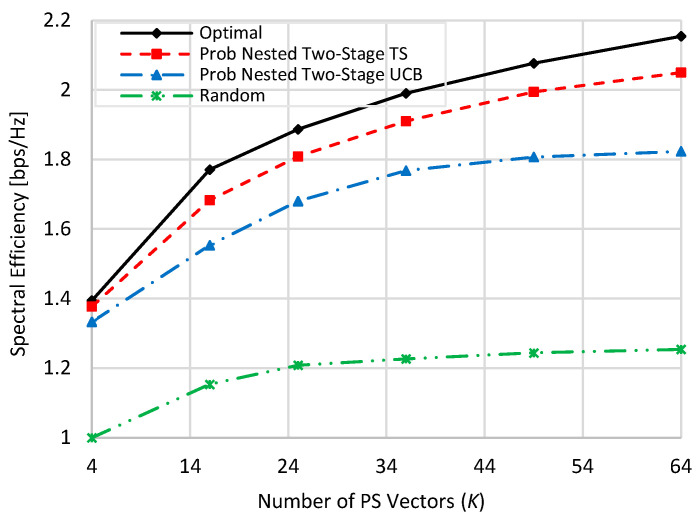
Spectral efficiency against the used number of PS vectors when *M = N = K* at 80% blockage.

Figure 3 shows the spectral efficiency of the schemes involved in the comparison at 80% blockage, where it simulates a harsh blockage environment. In this context, four paths out of the five channel paths between BS and RIS and between RIS and UE undergo blockage, including the LoS path. Compared to Figure 2, more than 50% decrease in spectral efficiency occurs in this harsh blockage environment compared to the zero blockage case. This is due to the low power received from the only surviving path out of the five paths. Despite this harsh blockage environment, the proposed two-stage MAB algorithms still show good spectral efficiency performance against the optimal one. Yet, the proposed two-stage TS outperforms other schemes due to the aforementioned reason. At *K* = 4, about 98.6%, 95.7%, and 72% of the optimal performance are obtained by the proposed nested two-stage TS, UCB, and random selection, respectively. These values become 95.35%, 84.4%, and 58.3% at *K* = 64, respectively. By comparing these ratios with those given in the previous paragraph and shown in Figure 2, it is clearly shown that the performance of random PS selection is highly degraded compared to the optimal performance due to the blockage effect. However, the proposed two-stage MAB algorithms almost have the same ratios of the optimal performance even in this harsh blockage environment. 

This means that the proposed algorithm can efficiently withstand the blockage effect due to its hypothesis of maximizing the achievable spectral efficiency irrespective of the environmental conditions.

**Figure 4:**
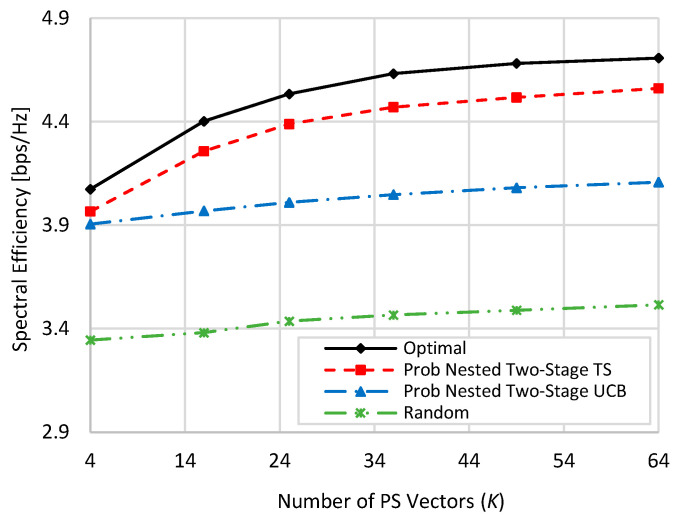
Spectral efficiency against the used number of PS vectors when *N =* 36 and *M =* 64 at zero blockage.

**Figure 5:**
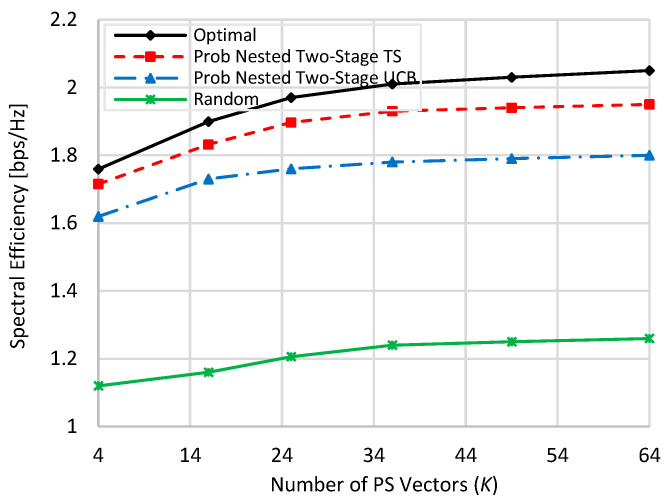
Spectral efficiency against the used number of PS vectors when *N =* 36 and *M =* 64 at 80% blockage.

Figure 4 and Figure 5 show the spectral efficiency performance of the compared schemes against the number of PS vectors (*K*) while *M* and *N* are different and fixed, where *N* = 36 and *M* = 64, at zero and 80% blockage, respectively. Again, the spectral efficiencies of all compared schemes are decreased under harsh blockage effect. By comparing Figure 4 with Figure 2 and Figure 5 with Figure 3, the spectral efficiencies of *K* = 4, 16, and 36 in Figure 4 and Figure 5 are slightly higher than those represented in Figure 2 and Figure 3, respectively. However, the spectral efficiencies of *K* = 49 and 64 in Figure 4 and Figure 5 are less than those given in Figure 2 and Figure 3 due to the use of a lower number of antenna elements at both BS and RIS. Typically, the half-power beamwidth is inversely proportional to the used number of antenna elements. Thus, increasing the number of antenna elements for generating the same codebook pattern, i.e., the same number of beams, has two opposite effects. On one hand, it generates narrower beams with larger antenna gains [12,13], and on the other hand, it is more vulnerable to phase shift errors. This increases the gain loss at the maximum gain [12] due to the increase in the interbeam null angles [13]. This is the reason why spectral efficiency is not highly increased when increasing the used number of antenna elements for generating the same number of PS vectors. Interested readers can check the detailed analysis given in [12,13] in this regard. Still, the proposed nested two-stage TS has the best performance that nearly matches the optimal performance due to the prementioned reasoning. At *K* = 4 and zero (80%) blockage, about 99% (97.4%), 97.5% (92%), and 83.5% (63.6%) of the optimal performance are obtained by the proposed nested two-stage TS, UCB, and random selection, respectively. These values become 97% (95%), 87% (87%), and 74.5% (61%) when *K* = 64, respectively. Again, the random selection is highly affected by the blockage effect more than the proposed nested two-stage MAB schemes.

**Figure 6:**
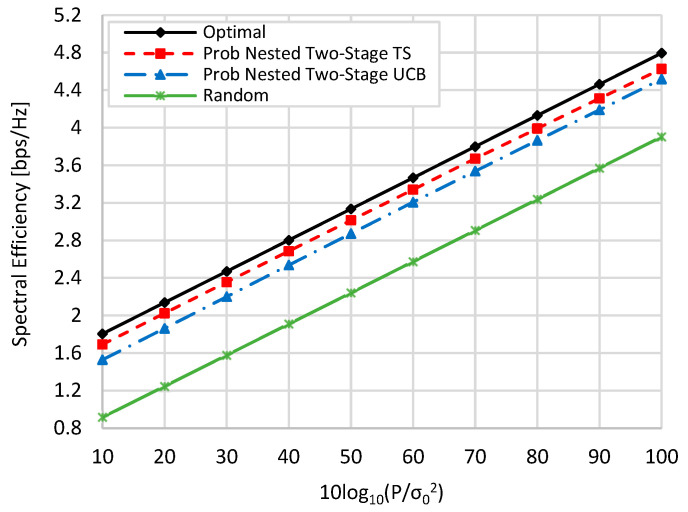
Spectral efficiency against TX SNR when *M = N = K =* 16 at zero blockage.

**Figure 7:**
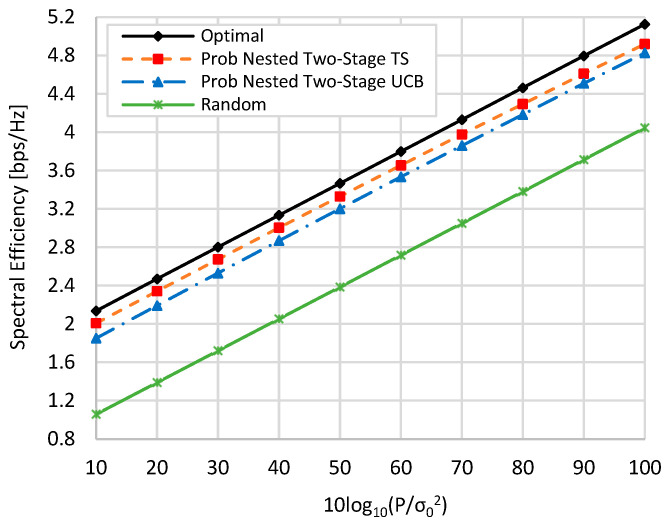
Spectral efficiency against TX SNR when *N* = 36, *M* = 64, and *K* = 16 at zero blockage.

Figure 6 and Figure 7 show the spectral efficiency against the TX signal-to-noise ratio (SNR), i.e., $10\log_{10}\left(\frac{P}{\sigma_{0}^{2}}\right)$, by changing the value of TX power P at zero blockage, when *K* = *M* = *N* = 16, and when *N* = 36, *M* = 64, and *K* = 16. Generally, as the value of P is increased, the spectral efficiency of all schemes is linearly increased. In addition, the spectral efficiencies in the case of *N* = 36 and *M* = 64 are slightly higher than those in the case when *N* = 16 and *M* = 16 due to the increase in beamforming gain resulting from increasing the number of antenna elements. From Figure 6 and Figure 7 and at TX SNR *=* 10 dB, about 93.8% (94%), 84.9% (86.9), and 51% (49.7%) of the optimal performance are obtained by the proposed nested two-stage TS, UCB, and random selection, respectively. These values become 96.5% (96%), 94.2% (94.16%), and 81.4% (78.8%) at SNR *=* 100 dB, respectively.

**Figure 8:**
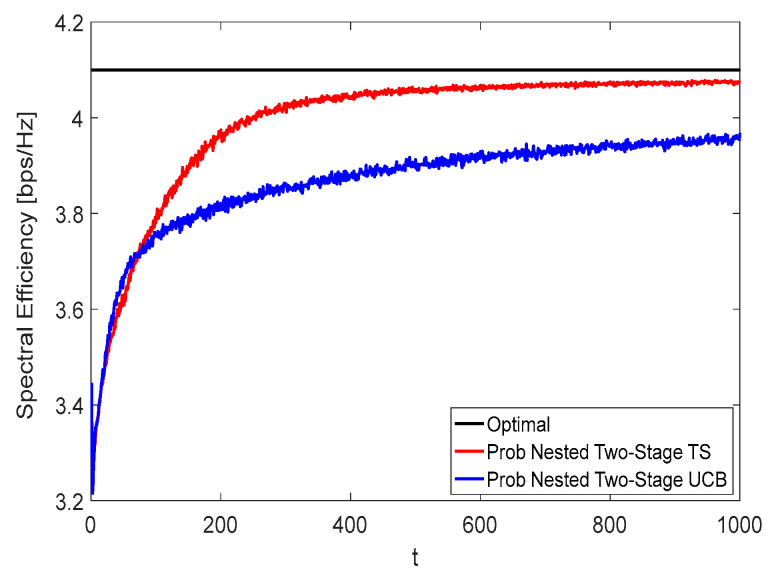
Spectral efficiency convergence at no blockage using *K* = *N* = *M* = 16.

**Figure 9:**
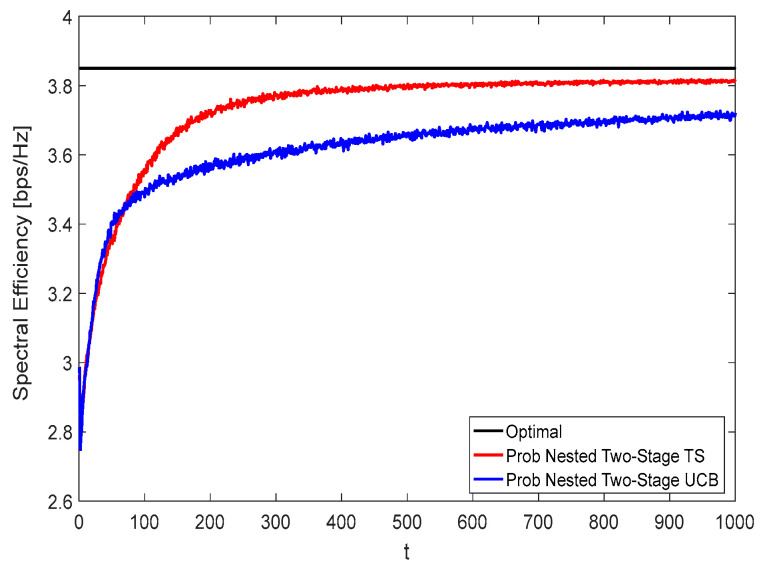
Spectral efficiency convergence at 50% blockage using *K* = *N* = *M* = 16.

Figure 8 and Figure 9 show the spectral efficiency convergence rate of the proposed nested two-stage MAB schemes against the time horizon t using *K* = *N* = *M* = 16 at zero and moderate blockage effect with blockage probability of 50%, respectively. Due to the effect of blockage, the spectral efficiency performance given in Figure 9 is lower than that represented in Figure 8. From these figures, it can be seen that the proposed nested two-stage TS has a faster convergence rate than the UCB-based one due to its Bayesian strategy. Interestingly, the proposed nested two-stage UCB has better convergence than the TS-based one at low values of t, where the TS algorithm starts to build up the prior/posterior distributions of the achievable reward. As these distributions are constructed, the TS converges faster than UCB, as shown by these figures. At t=400, about 99% (99%) and 95.12% (94.8%) of the optimal performance are obtained using the proposed nested two-stage TS and UCB-based one at zero (50%) blockage, respectively. 

**Figure 10:**
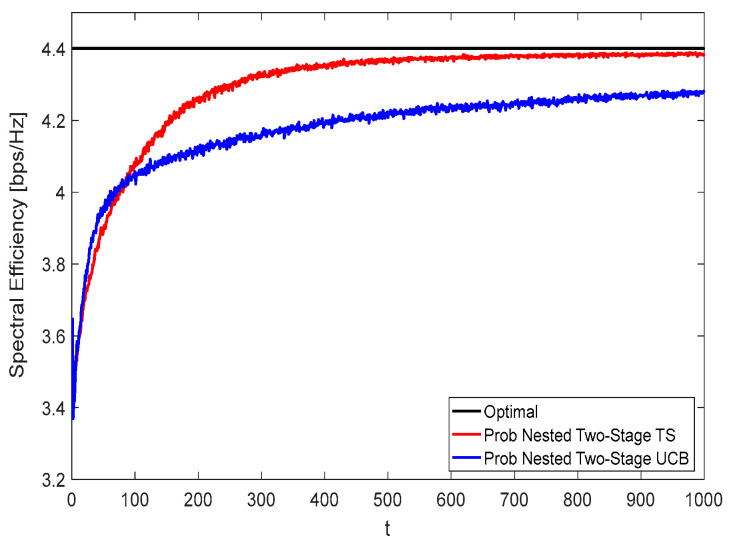
Spectral efficiency convergence at zero blockage using *N* = 36, *M* = 64, and *K* = 16.

**Figure 11:**
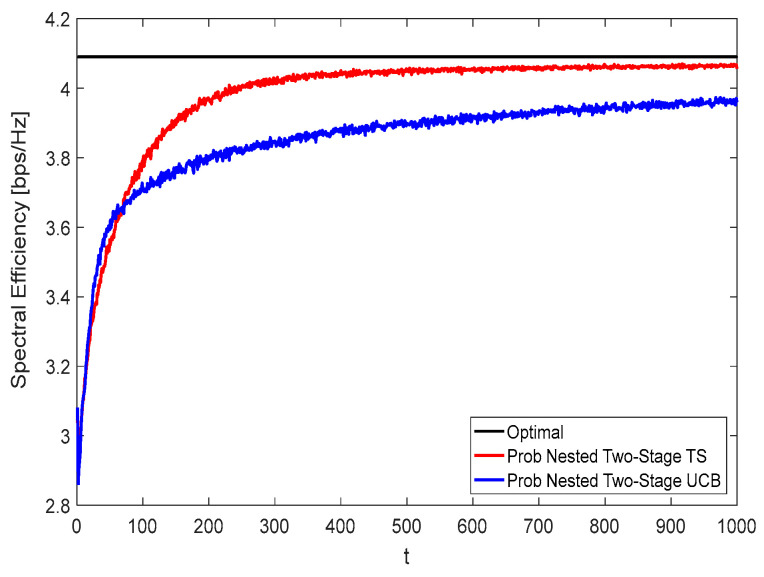
Spectral efficiency convergence at 50% blockage using *N* = 36, *M* = 64, and *K* = 16.

Figure 10 and Figure 11 show the spectral efficiency convergence rate using *N* = 36, *M* = 64, and *K* = 16 at zero and 50% blockage, respectively. By comparing Figure 8 with Figure 10 and Figure 9 with Figure 11, it is interesting to observe that the spectral efficiency and the convergence rate performances represented by Figure 10 and Figure 11 are better than those represented by Figure 8 and Figure 9, respectively. This comes from the increased number of antenna elements. At t=400, about 99.4% (99.1%) and 95.5% (95%) of the optimal performance are obtained using the proposed nested two-stage TS and UCB-based one at zero (50%) blockage, respectively.

**Figure 12:**
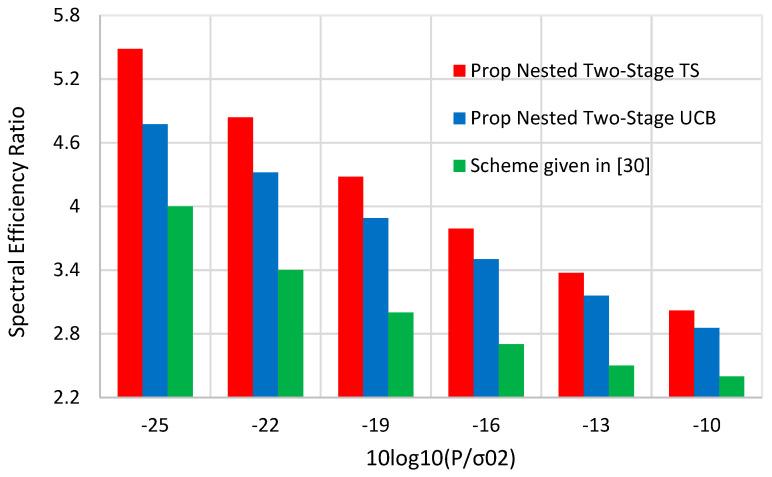
Spectral efficiency comparisons between the proposed nested two-stage MAB schemes and the scheme proposed in [30].

The suggested scheme of the perfect CSI-based approach presented in [30] reaches about 87~88% of the upper bound performance in the highest SNR scenario. This comes while assuming perfect mmWave CSI information, which is impractical in real scenarios. However, the proposed nested two-stage TS reaches about 94~99% of the optimal performance in the different simulation scenarios. Figure 12 shows the spectral efficiency ratio of the proposed nested two-stage TS, nested two-stage UCB, and the scheme proposed in [30] compared to the random performance against TX SNR. For fair comparisons, we used the same simulation parameters given in [30], i.e., *N* = 48, *M* = 100, and *K* = 6, and the same TX SNR values. As shown by this figure, the spectral efficiency ratio of the proposed nested two-stage TS has the best performance. In addition, both MAB schemes outperform the scheme presented in [30]. At SNR = −25 dB, the spectral efficiency ratios of the proposed nested two-stage TS, nested two-stage UCB, and the scheme given in [30] become 5.5, 4.8, and 4, respectively. This means that about 37.5% and 20% improvements in spectral efficiency performance are obtained by the proposed MAB schemes over the scheme presented in [30]. This comes without any need for knowing the CSI of both mmWave BS and RIS. 

The complexity analysis clearly shows that the proposed nested two-stage MAB scheme has low BT and computational complexities compared to the optimal solution. This is because the optimal strategy explores all available {ℛ,ℱ} pairs, which obtains its BT and computational complexities of order O(|ℛ||ℱ|). However, in the proposed MAB approach, the sets ℛ and ℱ are explored alternatively at every time t. Thus, the BT complexity of the proposed schemes is of order O(1). Regarding the computational complexities, for the proposed nested two-stage TS, the primary source of computational complexity comes from sampling a 1-dimensional Gaussian random variable and updating its related parameters with the complexity of O(|ℛ|+|ℱ|+1). In addition, the computational complexity of the proposed nested two-stage UCB comes from selecting the optimal PS and updating its corresponding parameters with the same computational complexity order of O(|ℛ|+|ℱ|+1). For example, when |ℱ|=36 and |ℛ|=64, the BT and computational complexities of the optimal solution are of order O(2304) while the BT and computational complexities of the proposed nested two-stage MAB approach will be O(1) and O(101), respectively. This means that about 99.96% and 96% reductions in BT and computational complexities are obtained, respectively. Consequently, the proposed nested two-stage MAB approach has a near-optimal performance with much lower complexity. 

## 6. Conclusions

In this paper, we have explored RIS-assisted mmWave communications. To avoid estimating the massive mmWave CSI at both RIS and UE, we proposed using antenna codebooks. Moreover, the problem of jointly optimizing the PS vectors at both mmWave BS and RIS was formulated as a MAB game, which contributes to relaxing the required BT overhead. In this context, a nested two-stage MAB strategy was suggested, and nested two-stage TS and UCB algorithms were proposed to implement the proposed strategy. Simulation analyses confirm the superior performance of the proposed two-stage TS compared to the UCB-based one. Moreover, the proposed nested two-stage MAB schemes outperform random selection and other benchmarks with a high convergence rate and low BT and computational complexities.

## Figures and Tables

**Table 1:** Simulation parameters.

Parameter	Value
P	10 dBm [2]
BW	2.16 GHz [2]
$L_{BR}$	5
$L_{RU}$	5
$T_{H}$	1000
d	λ/2
$\sigma_{0}^{2}$ (dBm)	−174 + 10log_10_(BW) + 10 [4]

## Data Availability

Not applicable.
